# Relemed: sentence-level search engine with relevance score for the MEDLINE database of biomedical articles

**DOI:** 10.1186/1472-6947-7-1

**Published:** 2007-01-10

**Authors:** Mir S Siadaty, Jianfen Shu, William A Knaus

**Affiliations:** 1 Department of Public Health Sciences, University of Virginia School of Medicine, Box 800717, Charlottesville, Virginia, 22908, USA

## Abstract

**Background:**

Receiving extraneous articles in response to a query submitted to MEDLINE/PubMed is common. When submitting a multi-word query (which is the majority of queries submitted), the presence of all query words within each article may be a necessary condition for retrieving relevant articles, but not sufficient. Ideally a relationship between the query words in the article is also required. We propose that if two words occur within an article, the probability that a relation between them is explained is higher when the words occur within adjacent sentences versus remote sentences. Therefore, sentence-level concurrence can be used as a surrogate for existence of the relationship between the words.

In order to avoid the irrelevant articles, one solution would be to increase the search specificity. Another solution is to estimate a relevance score to sort the retrieved articles. However among the >30 retrieval services available for MEDLINE, only a few estimate a relevance score, and none detects and incorporates the relation between the query words as part of the relevance score.

**Results:**

We have developed "Relemed", a search engine for MEDLINE. Relemed increases specificity and precision of retrieval by searching for query words within sentences rather than the whole article. It uses sentence-level concurrence as a statistical surrogate for the existence of relationship between the words. It also estimates a relevance score and sorts the results on this basis, thus shifting irrelevant articles lower down the list.

In two case studies, we demonstrate that the most relevant articles appear at the top of the Relemed results, while this is not necessarily the case with a PubMed search. We have also shown that a Relemed search includes not only all the articles retrieved by PubMed, but potentially additional relevant articles, due to the extended 'automatic term mapping' and text-word searching features implemented in Relemed.

**Conclusion:**

By using sentence-level matching, Relemed can deliver higher specificity, thus eliminating more false-positive articles. By introducing an appropriate relevance metric, the most relevant articles on which the user wishes to focus are listed first. Relemed also shrinks the displayed text, and hence the time spent scanning the articles.

## Background

MEDLINE^® ^is the U.S. National Library of Medicine's (NLM) primary literature database, indexing >15 million citations in the fields of medicine, nursing, dentistry, veterinary medicine, the health care system, and the preclinical sciences [1]. Encountering extraneous articles in response to a query submitted to MEDLINE/PubMed is not uncommon. However, every one of the articles retrieved contains all of the query words. This leads us to the conclusion that the presence of query words in an article is not a sufficient condition for the article to be relevant to user's query, although it is a necessary.

About 83% of queries sent to PubMed^®^, NLM's search engine for MEDLINE [2], are multi-word queries (see Additional File 1). When submitting a query with multiple words, the user is usually interested in some type of relationship [3] between the words, such that the "presence of relationship" between the query words in the article also becomes a necessary condition for relevance.

There are methods to ascertain the presence and type of relationship between two words in a text [4]. There are also numerous search engines, user interfaces, and software tools for retrieval of articles and information from MEDLINE [2, 5 to 14]. Table 1 lists some of them, but none of them detects either the presence or the type of relationship among the query words. Further research into these methods is needed before they can be implemented in the retrieval systems of MEDLINE.

Methods that eliminate increasingly more of the irrelevant articles will also tend to miss more of the relevant ones. Plus, as the total number of records in a database increases, it becomes increasingly hard to eliminate irrelevant articles without missing the relevant ones. Table 2 gives a scenario for a database with 16 million records (similar in size to MEDLINE). The search engine is assumed to work with 99% sensitivity (= recall, which is percentage of all relevant articles retrieved by the engine) and 99.99% specificity (percentage of all irrelevant articles eliminated by the engine); thus equivalent to an odds ratio of one million. Nevertheless, the majority of retrieved records (>76%) are irrelevant. One may be able to tune the search engine to increase the specificity even further (to 99.9999%), but it will decrease the sensitivity (to 50%), according to the theory of signal detectability [15,16]. This means that half of all relevant articles will be missed. To attain higher specificity without sacrificing sensitivity, the overall performance of the search has to increase.

In addition to trying to prevent irrelevant articles from appearing in the retrieved articles, one may also locate and isolate less relevant articles that have been retrieved. This can be done by estimating a relevance score for each retrieved article, and then sorting the articles by the score. Irrelevant retrieved articles will be shifted to the end of the list, effectively hidden from the user. Among the implemented information retrieval systems for MEDLINE, some do define relevance scores. However, these relevance scores are mainly based on place of occurrence and frequency of keywords extracted from the user's query. They do not incorporate the presence of a relationship between the query words.

We propose that if two words occur within an article, the probability that a relation between them is explained is clearly higher when the words occur within the same sentence (or adjacent sentences) versus remote sentences. This is a probabilistic expression of linguistic common sense. Therefore, sentence-level concurrence (co-occurrence) can be used as a surrogate for existence of the relationship between the words.

We have designed and implemented a publicly accessible search engine for MEDLINE. Our search engine, Relemed  [17], retrieves relevant articles by detecting sentence-level concurrence of search terms. The search engine estimates a relevance score where presence of the relationship between the words is an important component of the score. To maintain high sensitivity while increasing specificity, the search engine utilizes article-level concurrence as the last level of relevance. In this paper we explain the Relemed retrieval system and its relevance score, and compare it to PubMed.

## Implementation

Through a lease contract with NLM, we obtained MEDLINE data in extensible markup language (XML) format. We designed and implemented algorithms to extract title, abstract, and citation information from each XML article record, then scanned through the abstract text to detect and separate sentences. To detect a sentence we used '.', '?', and '!' as delimiters. We then joined back consecutive sentences where the period was sandwiched by single capital letters, some specific words such as 'etc.' and 'et al.', or by digits such as '0.05'.

We designed a database with two tables, to load the sentences. Table 3 shows the fields and their definitions. The first table of the database (table 3a) contains the sentences, the bulk of data, where an index is created for them. Field PMID (PubMed ID) is a unique integer number assigned by NLM to each article. Here we used PMID to link table 3a to table 3b. Field SNTNCID is equal to 1 for article title, and then 2 and bigger for abstract sentences. The second table of the database contains the citation information (author names, article title, journal name, publication date, issue and page numbers) for each NLM article. There is a many-to-one relationship between table 3a and table 3b. Table 3a is used to match user query to indexed articles, whereas table 3b is used to retrieve citation information for a given PMID.

We designed and implemented a software application to receive a user's query, prepare the query in SQL (structured query language), interrogate the database, format the database results in HTML language (HyperText Markup Language), and post it back to the user's browser.

Queries submitted to Relemed can simply be composed of one or a few words, separated by space. By default, the system uses Boolean 'and' operator to connect the words. Also, Boolean operators 'or' and 'not' are supported. One can use asterisk * for truncation, parentheses () for grouping, and quotes "" for exact phrase matching. These are in accordance with PubMed query language.

We used the Unified Medical Language System [18] to implement 'automatic term mapping'. When a query is submitted to Relemed, synonyms for query words are found and added automatically to the query, using 'or' as the operator, thus improving the sensitivity of the search.

**Figure 1:**
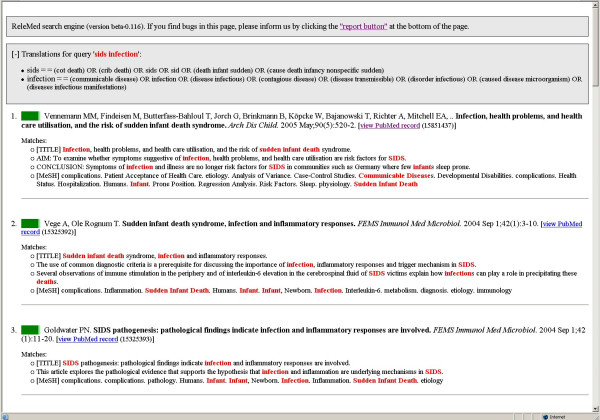
Format of search results returned by Relemed.

The system writes all the sentences matching the query in an HTML report, where the matched keywords are highlighted. The publication information for the article where the sentence was found is then added, as well as a hyperlink such that the user can easily navigate to the respective PubMed article, for potential drill down and for using features in PubMed that have not been implemented in Relemed. This format is shown in Figure 1.

We used freely available open source software to build the search engine, including Perl to pre-process data and write the query application [19], MySQL to implement the database [20], and Apache to serve the user's HTTP requests (HyperText Transfer Protocol) [21]. The server was installed with a Fedora operating system [22], hence the so-called LAMP architecture (Linux Apache MySQL Perl). XHTML (eXtensible HyperText Markup Language) was used to produce the user interface and the reports [23].

### Relevance metric

Given an article record, with title (one sentence), a few abstract sentences, and MeSH terms [24] (concatenated together and treated as one sentence), one can assign importance weights to each of the three sentence types (title, abstract, MeSH). Then one can combine the types to define several levels of 'relevance'. Thus one can try to measure how closely an article answers the user's query. Then one can sort the returned results by the relevance metric. This pushes the most relevant articles to the top of the result list, where the user would see the most relevant results first.

Table 4 defines eight relevance levels. Assuming user's query is 'word1 word2', in relevance level one, both the words should appear in title, and both words should appear in at least one sentence in abstract, and both words should appear in the MeSH terms, a stringent set of criteria. This we believe indicates that, in the majority of instances, the matched article would be of high relevance to the user's query, hence the first relevance level. The next levels are similarly defined, only the combinations of the types of sentences being different. Level 8 is different from the rest, as we first concatenate together all the sentences of an article, including title, all abstract sentences, and all the MeSH words. This makes one big 'sentence' from the whole article, which user's query is matched against. For example, word1 can be in the title, while word2 can be in MeSH words or in any of the abstract sentences (this is similar to PubMed's default). This level adds to the sensitivity of the search engine, thus reducing the probability of missing a relevant article. However level 8 has a low specificity, which is the reason we assigned the lowest relevance level to it.

### Evaluation method

We conducted two case studies to evaluate the Relemed search engine, and compare it to PubMed. The topics were chosen from real cases encountered in our daily practice. To decrease evaluation bias we concealed the source of each article (Relemed or PubMed) from the raters (who evaluated the biomedical relevance of the articles). This was accomplished by presenting the articles in a unified format to the raters. The two questions addressed were: Q1. Given a query, is the collection of articles returned by Relemed the same as PubMed? Q2.Are the most relevant articles listed at the top of the Relemed results?

Starting with a query, we chose a pre-defined article count *n*, like 10. We queried Relemed with the query, and saved PMIDs of the first *n *articles within each relevance level, hence giving a total of 8*n *PMIDs. Likewise we presented PubMed with the same query, and saved the first 8*n *PMIDs. Then we wrote a program into which we fed the two lists of 8*n *PMIDs. The program made a unique list of PMIDs. Then the program queried the database for each PMID, and wrote an HTML report where the article contents (all fields available under the 'MEDLINE' format, including title, abstract, and MeSH) are included. Keywords were highlighted in the HTML report, to facilitate evaluation process. Nothing in the report indicated which search engine (Relemed or PubMed) retrieved each article. Two raters inspected the articles independently, and assigned true positive (TP) or false positive (FP) labels to each, thus defining the 'gold standard'. To resolve potential discordance between the two raters, a discussion was made on each of the discordant articles to reach a consensus. Then the program transferred the TP and FP assignments back to the query results of each of the PubMed and Relemed, thus 'breaking the blind'. Finally we estimated the precision (= positive predictive value, which is percentage of retrieved articles that are relevant) for each of the relevance levels of Relemed, and consecutive bins of size *n *in PubMed.

To analyze the precision data, and to attach statistical significance (by constructing 95% confidence bands for the precision curves), we used 'local regression' implemented in package 'locfit' of R statistical language [25,26]. Also, to measure inter-rater agreement, we used Cohen's kappa, which measures the agreement between the evaluations of two raters when both are rating the same object.

In the Additional File #2 we present three more examples, further evaluating Relemed and comparing it to PubMed as benchmark.

## Results

### Case study 1: role of 'infection' in 'sudden infant death syndrome' (SIDS)

SIDS is death of an infant less than one year old that cannot be explained after thorough medical investigation [27]. Despite years of research, no definitive cause has been found, but there are many potential factors proposed by investigators, such as the position of baby during sleep, the use of a pacifier, history of parents' smoking, recent infection, change in temperature, etc. In this example the user wants to retrieve articles on SIDS that link infection as a potential cause of death in SIDS (or explains absence of such a relationship).

We used the query 'sids (infection or infect*)' in both PubMed and Relemed. We included the truncated word 'infect*' to automatically include all the variations of the word 'infect', such as infectious, infections, infective, etc. To include all other synonymous phrases (that do not necessarily contain the word 'infect'), we included the word 'infection'. This is necessary since the 'automatic term mapping' of the search engines only add synonyms for non-truncated words. We added the phrase '1900/1/1:2006/3/10 [dp]' to the query submitted to PubMed, to make the corpus of articles searched in the two search engines similar. This phrase limits "date of publication" to the range specified (March 10^th ^was the last date we updated Relemed database for the purpose of this study).

Both the engines searched all articles in MEDLINE from the earliest available publication dates to 3/10/2006. PubMed returned 608 articles, whereas Relemed returned 927. Twenty nine out of 608 articles of PubMed were not included in the Relemed results. These 29 articles were of two groups. Group one was articles with a publication date of 3/10/2006 or earlier, but added to the MEDLINE after March 10, 2006. Since this was the last date Relemed database was updated (for the purpose of this study), these articles did not exist in Relemed. The second group was articles where no variation or synonym for 'infection' existed in any field, but since PubMed 'explodes' a term to all of the narrower terms in the MeSH hierarchy tree under it, terms like 'septicemia' and 'septic abortion', as well as 'corneal ulcer' and 'trachoma', were included in the PubMed search but not Relemed. Of 927 articles returned by Relemed, 338 were not found by PubMed, for two reasons: 1. some synonyms for SIDS are not recognized by PubMed. An example is 'cot death'. This term was more common during 70's and 80's. 2. The acronym 'sids' in the submitted query is mapped to 'sudden infant death'. However in PubMed this longer phrase is only used to match to MeSH terms and not to abstract or title, thus missing some articles.

Table 5 shows count of articles in each Relemed relevance level. We used a cutoff of *n *= 10 to compose the PMID list. For levels where the total returned articles were smaller than 10, we used all available. This made a list of 74 PMIDs. We added the first 74 articles from PubMed, thus making a list of 148 PMIDs. Subsequently we omitted redundant PMIDs, and reduced the list to 111 unique PMIDs. The precisions were estimated by the method explained in the Evaluation section. The inter-rater agreement was 83% (19 discordant articles among the 111 unique PMIDs). The Kappa measurement of inter-rater agreement was 0.684, with a P-value of <0.001 (a Kappa of 1 indicates perfect agreement. A value of 0 indicates that agreement is no better than chance).

**Figure 2:**
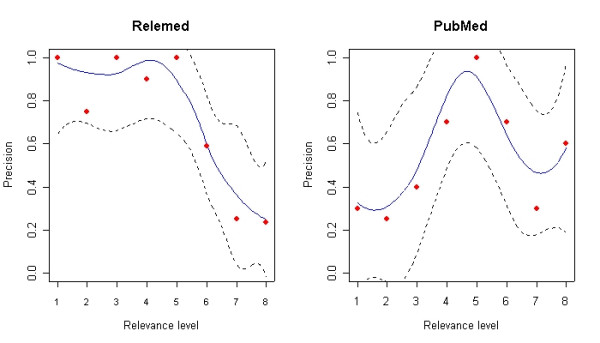
Trend of precision in Relemed versus PubMed for case study #1. The red dots show the observed precision in the 8 groups of PMIDs per search engine. The solid blue line is a fitted smoother curve for the observed binary data (true-positive versus false-positive). The dashed black curves are the estimated 95% global confidence bands.

Figure 2 shows the observed precision (the red dots) in the 8 groups of PMIDs per search engine. We fitted smoother curve (solid blue line) to the observed binary data (TP versus FP), to facilitate visualizing the trend. We also estimated 95% global confidence bands (the dashed black curves), for inference. Result pages in Relemed start with a precision of 100%, while the initial precision in PubMed is 30%. There is a decreasing precision trend in Relemed, but the trend in PubMed is not a monotone. One can draw decreasing lines (lines with negative slopes) for Relemed that are completely inside its 95% confidence band, but not for PubMed. On the other hand, one can draw horizontal lines within the 95% band of PubMed, but not Relemed. This suggests that the precision trends in the two search engines are significantly different. We note PubMed by default sorts the retrieved articles by reverse chronological order, which is not necessarily a relevance score. This supports the observation that PubMed results may attain their maximum precision anywhere along the list, and not always in the first page of results. The average precision in the first 74 articles of PubMed was 60.3%, while the estimated average precision for the first 74 articles of Relemed was 98.4%.

Table 6 shows an example of a false positive article. All instances of the query words in the article are highlighted and shown. Both 'infection' and 'SIDS' are mentioned in two separate sentences of abstract, plus the fact that both of them are in MeSH terms. However, no relation between the two is declared. This article belongs to relevance level #7 of Relemed and is #361 in the list of all articles. However, it is #41 in the PubMed result list (due to its publication date, which is the default sort of PubMed).

### Case study 2: finding 'questionnaires' for measuring 'health literacy'

Health literacy is the degree to which individuals have the capacity to obtain, process, and understand basic health information and services needed to make appropriate health decisions [28]. In this example, the user has a research project in which he wants to measure health literacy of the participants. He is interested in finding publications that give clues about existing questionnaires/instruments for health literacy.

We used the query

"health literacy" and (instrument* or question* or measur* or scale* or assessment* or index* or test*)

and PubMed returned 157 articles, whereas Relemed returned 158 of which 153 were shared with PubMed (a 96.8% overlap). There were 4 articles in PubMed that were absent from Relemed. All the four were articles with publication dates within the studied range (from the earliest publication date to 3/10/2006), but that have been added to the MEDLINE after March 10, 2006 (the last update for Relemed database). The five articles found by Relemed but not by PubMed contained the term 'health literacy' and 'test' in abstract or title, but still could not be retrieved by PubMed. These seem to be false negatives for PubMed.

**Figure 3:**
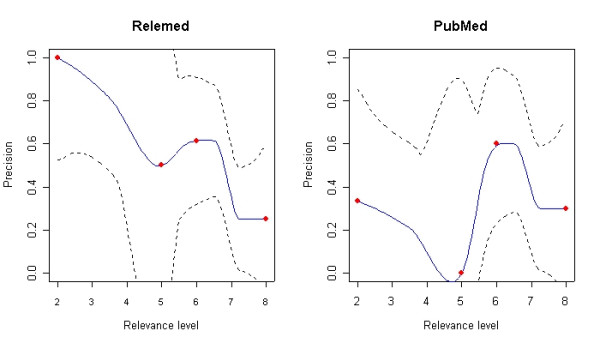
Trend of precisions for case study #2. The red dots show the observed precision in the 8 groups of PMIDs per search engine. The solid blue line is a fitted smoother curve for the observed binary data (true-positive versus false-positive). The dashed black curves are the estimated 95% global confidence bands.

In Figure 3 the precision starts from a much higher point (100%) in Relemed compared to PubMed, and shows a decreasing trend. Note that the 95% confidence bands are rather wide in this case study, mostly due to the small number of articles per relevance level.

The precision in PubMed for the first 28 articles was 39.3%, while precision for the first 28 articles of Relemed was estimated at 68.9%. The Kappa measure of inter-rater agreement was 0.496, which was significantly higher than chance (P-value < 0.001).

## Conclusion

### Comparison of information retrieval systems of MEDLINE

There are more than 30 retrieval services that use MEDLINE as their data source [29], some of which are shown in Table 1. Some use MEDLINE as the main or the only data source, such as PubMed, OVID, SLIM, askMEDLINE, and eTBLAST. Others use multiple databases, e.g. MedMiner. Some return articles as their main results (PubMed), while others return some digested form, such as a graph (Chilibot and ConceptLink). Some focus on data-mining, MedBlast and HAPI (High-density Array Pattern Interpreter); others on genomics or proteomics (GoPubMed and iHOP). Some are designed for "literature-based discovery", finding relationships between biomedical concepts from MEDLINE that are not expressed in any article directly, e.g. Arrowsmith and BITOLA. Some are specialized in the classification of articles, e.g. AnneOTate, CISMeF, and MedMOLE.

The majority of these services do not estimate relevance scores. None of them incorporate any relationship between the words in computing the relevance score.

OVID supports a 'proximity operator' where the user can ask for the two keywords to be within some specified distance (measured by the number of words separating them). However, this feature does not recognize sentence boundaries. For example, a word at end of a sentence is considered adjacent to the word in the beginning of the next sentence, and is treated the same way as when the two words were adjacent within the same sentence. Moreover, there is no automatic feature to utilize the adjacency operator, for sorting the resulting articles by increasing distance between the keywords matched per article. The user has to manually submit multiple queries with increasing proximity distances to be able to have a gradient of distances. Also note that word-proximity has less obvious cut-off values, compared to 'sentence' which is a more clear-cut linguistic unit.

PubMed has a feature called "Related Articles". After a search retrieves some articles, each article has a link that displays 'related articles' to it. These related articles in turn are sorted by a relevance score [30]. However, this score does not incorporate the original query that the user submitted. In other words, given that many biomedical concepts can be expressed in an article, the article can be retrieved by very different queries sent by different users. However, in all these instances the related articles of the original article are exactly the same, irrespective of what concept the user was originally interested in. PubMed also gives the options to sort the search results by one of the four criteria: 1.Pub Date 2.First Author 3.Last Author 4.Journal. These do not necessarily reflect the relevance of an article to the user's query.

One may try to use some of the PubMed features to detect 'relation' between words for a multi-word query. Three methods come to mind: 1.One can limit the search to the titles only. Then if the (two) words appear in the title, it has a high probability that some sort of relation is declared between them in the article. Although this method could attain fairly high specificity, it may miss relevant articles because it does not utilize any of the sentences of the abstract, i.e. it is potentially of low sensitivity. 2. If the two or more words the user is asking have hierarchical relation in the MeSH, then MeSH can show high specificity. For example, when the user is interested in adverse effects of antidepressant therapy, the MeSH subheading 'adverse effects' to the MeSH heading 'antidepressive agents' is a good query. A similar case is when all the query words map to a single MeSH term. For example, query 'two dimensional gel electrophoresis' maps to "electrophoresis, gel, two-dimensional" [MeSH Terms]. In such cases many of the retrieved articles can be relevant. 3.If the query words are mainly used consecutively in the article text, one may be able to use quoting (the operator ""), in order to instruct PubMed to retrieve articles where the words appear exactly (in the same proximity and order) as they are in the quoted phrase. However, these are not common cases.

### Relemed

We emphasized that the majority of queries sent to MEDLINE/PubMed are multi-word queries, where two or more words are included in the query. For these queries, the user can be looking for articles that are about 1.each word, and 2.some relationship between the words. Currently, the retrieval systems of MEDLINE (including PubMed) identify articles with the requested words but not their relationship. Drawing on linguistics, the chance of the article claiming some relation between the two words is higher when they concur within a sentence than an article (or abstract). This was the basis for creating the Relemed search engine.

There is a limit to the amount of text a user is willing or able to scan. By using a sentence level matching, Relemed is able to deliver higher specificity, thus reducing false positive (FP) articles. Also, by introducing relevance metric, the most useful articles are shown first, where the user focuses most. By composing the matching sentences and highlighting the keywords, Relemed shrinks the text and the time the user spends for the 'scan & eliminate' process (where the user reads the titles or quickly scans the abstracts, and decides whether to eliminate the article or leave it for the next round of more in-depth screening). The two examples used in the paper demonstrated that the higher precision attained at the start of results in Relemed facilitates this type of screening.

Recognizing, however, that Relemed returns almost identical collection of articles as PubMed, one question is the location of the false positive articles in the Relemed results? We believe that Relemed's relevance levels 7 and 8 would contain majority of FPs.

There are limitations in the current implementation of Relemed. First, we note that since Relemed matches a query against each single sentence, having too many words in the query might return no article in the first few relevance levels. And second, if the total number of articles returned from MEDLINE is small, sorting them according to a relevance metric may not improve the retrieval process significantly.

There are also additional features that can be added to Relemed to improve its usefulness. For example, it would be helpful if Relemed showed the total number of matched articles per relevance level in the first page of results. The software currently used to implement Relemed does not support a fast response time for such a feature. It would also be useful to add search capability for fields like author names, and publication dates. We have also considered making the matched sentences and the article contents collapsible/expandable (via JavaScrips for example), rather than showing all the material at once. Finally, it may be possible to refine the relevance score by utilizing natural language processing algorithms in ascertaining the relation between words.

Additional evaluation and comparison of Relemed with PubMed and other search engines is essential. But we believe these initial results hold promise for improving the precision and efficiency of search using the sentence-level search capabilities and relevance sorting.

## Availability and requirements

Project name: Relemed

Project home page: 

Operating systems: Platform independent

Programming language: Perl

Other requirements: None

License: Free access

Any restrictions to use by non-academics: Incorporation into commercial products restricted

## Abbreviations

FP: False Positive

HTML: HyperText Markup Language

HTTP: HyperText Transfer Protocol

LAMP: Linux Apache MySQL Perl

MeSH: Medical Subject Headings

PMID: PubMed ID

Relemed: Sentence-level search Engine with Relevance score For MEDline

SIDS: Sudden Infant Death Syndrome

SQL: Structured Query Language

TP: True Positive

XHTML: eXtensible HyperText Markup Language

XML: eXtensible Markup Language

## Competing interests

A patent application has been filed by MSS and WAK. JS declares no competing interest.

## Authors' contributions

MSS conceived of the method, carried out its implementation, participated in its evaluation, and drafted the manuscript. JS participated in the evaluation and drafting the manuscript, and gave feedback to improve the search engine. WAK participated in drafting the manuscript, and gave feedback to improve the search engine. All authors read and approved the final manuscript.

## Pre-publication history

The pre-publication history for this paper can be accessed here:



## Supplementary Material

Additional File 1 explaining the analysis we have done on all the queries received by NLM's PubMed in one day. Here we derive percentage of multi-word queries. Click here for file

Additional File 2 presenting three more examples to evaluate performance of Relemed, and compare with PubMed as the benchmark. Click here for file

## Figures and Tables

**Table 1:** Examples of retrieval services for MEDLINE

**service**	**availability**	**relevance score**	**description**
PubMed	public/free	no	NLM's search engine for MEDLINE
SLIM	public/free	no	alternative search interface using slider controllers to implement search limits, methodology filters, and MeSH terminologies
askMEDLINE	public/free	no	free-text, natural language query tool for PubMed
eTBLAST	public/free	yes	inputs an entire paragraph and returns articles that are similar to it
Ovid's MEDLINE	subscription required	no	a search engine to MEDLINE
HubMed	public/free	yes	shows first the articles that contain the search terms most frequently in the title and/or abstract
PubMedAssistant	public/free	no	biologist-friendly interface for enhanced PubMed search
CISMeF	public/free	no	gives ranked list of relevant specialties that relate to topics discussed in each article
GoPubMed	public/free	no	classifies the retrieved articles using Gene Ontology terms
AnneOTate	public/free	no	A tool for summarizing the results of a PubMed query
ArrowSmith	public/free	no	A tool for identifying links between two sets of Medline articles
PubMed Gold	public/free	no	finds PDFs for PubMed citations

**Table 2:** Tuning a search engine to attain two different scenarios of retrieval.

Scenario 1. Query with specificity of 99.99% is insufficient for a database of 16 million records.				
		The truth		

		relevant records	irrelevant records	

search engine	records returned to user	495	1,600	2,095
	records eliminated	5	15,997,900	

		500	15,999,500	16,000,000

odds ratio	1,000,000.00			
Specificity	99.99%			
sensitivity (recall)	99.01%			
Precision	23.63%			

Scenario 2. The price for a very high specificity: Missing a large number of relevant records.				

		The truth		

		relevant records	irrelevant records	

search engine	records returned to user	250	16	266
	records eliminated	250	15,999,484	

		500	15,999,500	16,000,000

odds ratio	1,000,000.00			
Specificity	99.9999%			
sensitivity (recall)	50.00%			
Precision	93.99%			

**Table 3:** Database tables, and their fields

**Database table 3a**		
**Field**	**Description**	**Indexed**
PMID	PubMed ID number	no
SNTNCID	sentence ID number	no
Sentence	text of the sentence	yes

**Database table 3b**		

**Field**	**Description**	**indexed**
PMID	PubMed ID number	yes
Citation	Citation information for the article	no

**Table 4:** The eight relevance levels defined by Relemed.

**Relevance level**	**Query must match**
1	T and A and M
2	T and A
3	T and M
4	A and M
5	T
6	A
7	M
8	TAM

T = title

A = at least one abstract sentence

M = concatenated MeSH terms

TAM = title, abstract, and MeSH concatenated into one sentence

**Table 5:** Count of articles in each Relemed relevance level for the two case studies

	**Count of retrieved articles**	
**Relevance**	**Case study #1**	**Case study #2**

L1 T&A&M	32	0
L2 T&A	4	6
L3 T&M	36	0
L4 A&M	78	0
L5 T	12	2
L6 A	182	68
L7 M	290	0
L8 TAM	257	82

Total	891	158

**Table 6:** A false positive article for query of case study #1, where query words do concur, both in text and in MeSH (but not in the same sentence).

DiFranza JR, Aligne CA, Weitzman M. **Prenatal and postnatal environmental tobacco smoke exposure and children's health**. *Pediatrics*. 2004 Apr;113(4 Suppl):1007-15. (PMID 15060193) ... A large literature links both prenatal maternal smoking and children's ETS exposure to decreased lung growth and increased rates of respiratory tract **infection**s, otitis media, and childhood asthma, with the severity of these problems increasing with increased exposure. **Suddeninfantdeath**syndrome, behavioral problems, neurocognitive decrements, and increased rates of adolescent smoking also are associated with such exposures. ... [MeSH] drug effects. etiology. adverse effects. Animals. Asthma. etiology. Child. Child Behavior. drug effects. Embryonic and Fetal Development. Female. Humans. **Infant**. Intelligence. drug effects. Otitis Media. etiology. Pregnancy. Respiratory Tract **Infection**s. Smoking. adverse effects. **SuddenInfantDeath**. etiology. Tobacco Smoke Pollution. analysis
