# Supplementary material for: Relemed: sentence-level search engine with relevance score for the MEDLINE database of biomedical articles
Source: BMC Med Inform Decis Mak. 2007 Jan 10;7:1. doi: 10.1186/1472-6947-7-1 (PMC1780044; doi:10.1186/1472-6947-7-1)
Supplement: Additional File 1 — explaining the analysis we have done on all the queries received by NLM's PubMed in one day. Here we derive percentage of multi-word queries. [file 1472-6947-7-1-S1.pdf]

Estimating number of words per query in queries submitted to NLM's PubMed.

We took one day worth of all queries submitted to NLM's PubMed [taken from ftp://ftp.ncbi.nih.gov/toolbox/pubmed/query-logs/ as of June 2006]. There were 2,995,234 queries. Then we wrote a computer script to go through each query and split it into words. The split function used white-space as the delimiter to separate the words. The script also detected presence and count of Boolean operators AND and OR in each query. Finally it computed count of (non-operator) words in each query. This table shows percentage of queries with different word counts.

number of words in query	percentage of total submitted queries
0	2.60
1	14.51
2	37.67
3	21.01
4	11.65
5	5.09
6	2.66
7	1.31
8	0.83
9	0.57
10+	2.08

Apparently there are times when a user clicks the submit button without typing any words in the search box (we checked and this figure is not a computational error of the script).

There are 14.5% single-word queries. The rest of the queries (82.9%), the majority of them, are multi-word queries.

We note that within multi-word queries, there are queries where the whole query maps to a single MeSH term. For example, query 'two dimensional gel electrophoresis' maps to "electrophoresis, gel, two-dimensional"[MeSH Terms]. In such cases many of the retrieved articles can be relevant. However, this is not a common case. For the majority of multi-word queries, ascertaining presence of relation between the words in an article will improve the relevance score.
