# Supplementary material for: Relemed: sentence-level search engine with relevance score for the MEDLINE database of biomedical articles
Source: BMC Med Inform Decis Mak. 2007 Jan 10;7:1. doi: 10.1186/1472-6947-7-1 (PMC1780044; doi:10.1186/1472-6947-7-1)
Supplement: Additional File 2 — presenting three more examples to evaluate performance of Relemed, and compare with PubMed as the benchmark. [file 1472-6947-7-1-S2.pdf]

Evaluating articles retrieved by Relemed, using PubMed as the benchmark

It is hard to draw definitive conclusions about performance of Relemed by only two case studies presented in the paper. On the other hand, it is infeasible to evaluate Relemed by a “representative sample” of the queries sent to MEDLINE/PubMed (due to their number and variety).

In this document we present a series of examples, further studying performance of Relemed, using PubMed as the benchmark. Here we perform a less formal comparison (compared to the two case-studies presented within the paper), with no “blinding”, or independent ratings. We confine ourselves to the first 10 articles per search engine, so no thorough examination and cross-comparison of all the articles each engine retrieves.

To build queries for the examples, we chose to locate and use biomedical concepts “accidentally” mentioned by the reviewers of our paper in their critique. We say accidentally because it was not the reviewers’ intended use of the concepts (we are trying to show that we have not “hand-picked” any specific concept or query for the comparisons). We believe by reading this document the reader would be able to choose his own biomedical concepts and try them on the two engines, and repeat the process several times; hence a first-hand experience with the strengths and pitfalls of Relemed.

Here we present three additional examples. Example 1 is on temperature and heart failure. Example 2 is about infection and celiac disease. And finally example 3 is on role of radiation in Down syndrome.

The following table shows the summary of the three examples.

Precision of the first ten articles per search engine per example.

	precision (positive predictive value)	
Example	PubMed	Relemed
1. Heart failure and temperature	40%	90%
2. Celiac disease and infection	20%	70%
3. Down syndrome and radiation	30%	60%

Example 1. Relation between temperature and heart failure

We used the query ‘chf temperature’ for this example. We added phrase ‘1900/1/1:2006/3/10[dp]’ to the query for PubMed, for all of the examples of this document (since March 10, 2006 was the last date Relemed database was updated for the purpose of this study). The purpose of this example is to find out if there is any link or relationship between congestive heart failure (CHF) and temperature (body, air, or other kinds of temperature). Note we are interested in papers claiming any type of relation, direct or inverse, or even papers claiming lack of relation (collectively considered as the relevant papers). Papers are considered irrelevant if they contain the concepts ‘chf’ and ‘temperature’ but say nothing about presence or absence of any kind of relationship between the two.

The following is the first ten articles returned by PubMed, and then the first ten articles returned by Relemed. We rated the relevance of the PubMed ten articles as 0, 0, 0, 0, 1, 1, 0, 1, 1, 0 (1 for relevant, and 0 irrelevant), and the relevance of the Relemed ten articles as 1, 1, 1, 1, 1, 1, 1, 1, 0, 1. Thus, there are 4 relevant articles in PubMed and 9 in Relemed, 40% versus 90%.

Example 2. Relation between infection and celiac disease

In this example we want to see if there is any evidence in the published articles linking infection and celiac disease. We used the query ‘celiac disease infection’.

We rated the relevance of the PubMed first ten articles as 0, 1, 1, 0, 0, 0, 0, 0, 0, 0 (1 for relevant, and 0 irrelevant), and the relevance of the Relemed ten articles as 1, 1, 0, 0, 0, 1, 1, 1, 1, 1. Therefore, there are 2 relevant articles in PubMed and 7 in Relemed, 20% versus 70%.

Example 3. Role of radiation in Down’s syndrome

Here we sought articles that link radiation to Down’s syndrome. We used the query ‘down syndrome radiation’. We rated the first ten articles retrieved by PubMed as 0, 0, 1, 0, 0, 0, 0, 1, 0, 1. The top ten articles for Relemed were rated as 1, 1, 1, 0, 0, 0, 1, 1, 0, 1. Therefore 30% versus 60% relevance in PubMed versus Relemed.

Note, we did not reproduce the abstracts here, due to copyright issues.
